# Therapeutic Potential of Mesenchymal Stem Cell–Derived Secretome in Bacterial Infections: Bridging Acute Inflammation and Biofilm‐Associated Diseases

**DOI:** 10.1155/ijm/4040189

**Published:** 2026-11-09

**Authors:** Mohamad Arif, Agung Putra, Setyo Trisnadi, Sri Priyantini Mulyani

**Affiliations:** ^1^ Doctoral Program in Biomedical Science Faculty of Medicine Universitas Islam Sultan Agung Semarang Indonesia unissula.ac.id; ^2^ Department of Internal Medicine Faculty of Medicine Universitas Islam Sultan Agung Semarang Indonesia unissula.ac.id; ^3^ Stem Cell and Cancer Research (SCCR) Semarang Indonesia; ^4^ Lecturer Biomedical Science Undergraduate Program Agung Putra University (APU) Semarang Indonesia; ^5^ Department of Paediatrics Faculty of Medicine Universitas Islam Sultan Agung Semarang Indonesia unissula.ac.id

**Keywords:** bacterial persistence, biofilm-associated infection, host-directed therapy, MSC-derived secretome, persister cells

## Abstract

Incomplete bacterial eradication despite appropriate antimicrobial therapy contributes to the progression of some bacterial infections toward chronic or recurrent disease. Beyond genetically encoded antimicrobial resistance, bacterial persistence may be sustained by phenotypically tolerant subpopulations, including persister cells, together with biofilm‐associated growth. These states arise within heterogeneous microenvironments shaped by antimicrobial exposure, immune pressure, nutrient and oxygen limitation, and tissue injury. Failure of inflammation to resolve effectively may further promote persistent inflammatory signaling, impaired phagocytic function, localized hypoxia, and extracellular matrix disruption, thereby creating conditions that favor bacterial survival and biofilm stability. This review critically examines the mesenchymal stem cell–derived secretome as a potential host‐directed adjunct for persistent and biofilm‐associated bacterial infections. Rather than reiterating its antimicrobial, immunomodulatory, and regenerative properties, we integrate direct antibiofilm and antimicrobial evidence with indirect host‐mediated mechanisms involving NF‐*κ*B modulation, proresolving macrophage responses, efferocytosis, and tissue repair. These actions may converge on host–pathogen niches that sustain bacterial persistence. However, direct evidence that MSC‐secretome reduces persister frequency, reverses bacterial dormancy, or eliminates persister populations remains limited. By distinguishing experimentally demonstrated effects from mechanistically inferred actions, this review proposes a host–pathogen ecological framework for evaluating MSC‐secretome in bacterial persistence. Current evidence remains predominantly preclinical. Key priorities include improved product standardization and potency assessment, validation in physiologically relevant chronic infection models, and clearer definition of dosing, delivery, and combination strategies with conventional antimicrobials.

## 1. Introduction

Bacterial infections continue to represent a major contributor to global morbidity and mortality, not only through their acute manifestations but also through their propensity to evolve into chronic or recurrent disease states [1]. Although advances in antimicrobial therapy have substantially reduced mortality during the acute phase, numerous clinical challenges remain evident in daily practice. Recurrent pneumonia, nonhealing wounds, device‐ and implant‐associated infections, and persistent urinary tract infections illustrate that bacterial eradication is often incomplete [2, 3]. These conditions are frequently characterized by repeated episodes of infection, sustained inflammation, progressive tissue damage, and increasing healthcare burdens [4, 5]. Collectively, these patterns underscore the limitations of conventional therapeutic strategies that rely primarily on pathogen killing, highlighting the need for a more comprehensive understanding of the biological processes that enable bacterial survival and persistence despite appropriate antimicrobial interventions [4–7].

Under physiological conditions, acute bacterial infections are typically controlled through a well‐coordinated inflammatory response. This process is predominantly mediated by the innate immune system, which rapidly recruits neutrophils, activates tissue‐resident macrophages, and induces the release of proinflammatory mediators that restrict pathogen growth and facilitate microbial clearance [8–10]. When these defense mechanisms function effectively, the acute inflammatory phase transitions into a regulated resolution program, followed by tissue repair and regeneration. Through this sequence, structural integrity, biological function, and immune homeostasis are ultimately restored [11–13].

However, in many clinical scenarios, this ideal sequence of immune events fails to occur. Excessive bacterial burden, impaired phagocytic function, hypoxic or structurally damaged tissue microenvironments, and delays or inadequacies in antimicrobial therapy can collectively compromise the host′s ability to eliminate pathogens effectively [14]. Under such selective pressures, a small subset of bacteria may adapt by entering a transient, highly tolerant state known as persister cells. These cells exhibit markedly reduced metabolic activity and do not possess genetic mutations associated with antibiotic resistance, yet they survive antimicrobial exposure and immune attack because most conventional drug targets are minimally active [15]. Although persister cells do not provoke a pronounced acute inflammatory response, their continued presence sustains low‐grade immune stimulation, disrupts the resolution phase, and ultimately delays full tissue recovery [16].

As infection persists, surviving bacteria often reorganize into biofilms as a collective protective strategy. Biofilms represent highly structured microbial communities embedded within an extracellular matrix that creates a heterogeneous microenvironment with gradients in oxygen availability, nutrient distribution, and antimicrobial penetration [17]. This architectural complexity not only limits the access of phagocytic cells and reduces antibiotic effectiveness but also supports the maintenance of persister cells and preserves phenotypic heterogeneity within the bacterial population. Through these characteristics, biofilms function as an ecological niche that facilitates the transition from incomplete clearance of acute infections to chronic and relapsing disease states that are notoriously difficult to manage clinically [18, 19].

The limitations of conventional therapeutic approaches in achieving comprehensive control of bacterial infections highlight the need for alternative strategies that extend beyond direct pathogen eradication [20, 21]. Within this context, the mesenchymal stem cell–derived secretome (MSC‐secretome) has emerged as a biologically compelling candidate with multifaceted therapeutic potential. MSC‐secretome exhibits immunomodulatory, anti‐inflammatory, and indirect anti‐infective activities, positioning it as a promising tool to address therapeutic gaps that remain unmet by antibiotics alone [22]. This perspective suggests that future infection management should move beyond the pursuit of new antimicrobial agents and instead incorporate secretome‐based approaches capable of modulating host responses, controlling infection, and simultaneously supporting tissue repair and regeneration [23].

Given the complex pathogenesis of bacterial infections—particularly the roles of biofilms and persister cells in therapeutic failure—there is a growing need for treatment frameworks that extend beyond direct antibacterial targeting. Effective strategies must also be capable of modulating host immune responses and disrupting the biological microenvironments that sustain pathogen persistence [24].

Within this context, host‐directed therapeutic approaches have gained increasing relevance. The MSC‐secretome, which contains a diverse array of soluble factors and extracellular vesicles with immunomodulatory, proresolutive, and regenerative properties, offers a biologically grounded therapeutic platform [25]. Rather than acting through direct bactericidal mechanisms, MSC‐secretome has the potential to contribute to dismantling the tolerogenic niches that support bacterial persistence and biofilm stability, thereby bridging the gap between the resolution of acute inflammation and the management of chronic, biofilm‐associated infections [20, 26].

Building on this background, the present review examines the therapeutic potential of MSC‐secretome in bacterial infections by reframing failed bacterial eradication as a problem of infection biology that emerges from dynamic host–pathogen interactions, rather than as a limitation of antibiotic efficacy alone [24]. Persister cells and biofilms are considered phenotypic tolerance states and microbial ecological adaptations shaped by oxygen and nutrient gradients, protection afforded by the extracellular polymeric substance (EPS) matrix, and sustained immune pressure, providing a mechanistic basis for infection recurrence in the absence of genetically encoded antimicrobial resistance [5]. Previous reviews have summarized the antimicrobial properties of MSCs and their secretomes, including their potential relevance to bacterial persisters [22, 25, 27], whereas recent systematic reviews have evaluated preclinical antibacterial evidence across MSC sources and derivatives [28]. The present review extends these perspectives by integrating persister biology, biofilm ecology, failed inflammatory resolution, and tissue microenvironmental remodeling within a host–pathogen ecological framework. Importantly, we distinguish experimentally demonstrated antimicrobial and antibiofilm effects from host‐mediated mechanisms that remain inferential with respect to persister biology.

## 2. When Acute Inflammation Fails

In the early phase of bacterial infection, the innate immune system plays a central role through the activation of pattern recognition receptors (PRRs), which detect pathogen‐derived molecules and signals of cellular damage [8]. PRRs encompass several major families, including toll‐like receptors (TLRs) and nucleotide‐binding oligomerization domain‐like receptors (NLRs), each responsible for recognizing distinct classes of pathogen‐associated molecular patterns (PAMPs) and damage‐associated molecular patterns (DAMPs) [10, 29]. TLRs located on the cell surface and within endosomes sense microbial components such as lipopolysaccharides, lipoproteins, and nucleic acids, subsequently engaging adaptor proteins such as MYD88 and TRIF to initiate downstream signaling via NF‐*κ*B and MAPK pathways [8, 10]. These coordinated signaling events ensure rapid detection of bacterial invasion and orchestrate the production of proinflammatory cytokines and chemokines that drive the recruitment of neutrophils and macrophages to the site of infection [10, 30].

Upon activation of the NF‐*κ*B pathway, the p65/RelA subunit translocates into the nucleus and initiates the transcription of a broad array of proinflammatory genes. These include key cytokines such as TNF‐*α*, IL‐1*β*, and IL‐6, along with various chemokines that coordinate the recruitment of neutrophils and macrophages to sites of infection [31]. Through these actions, NF‐*κ*B occupies a central position in linking pathogen recognition by the innate immune system to the production of inflammatory mediators that shape the magnitude of the acute response to bacterial invasion [30, 32].

During the acute phase of bacterial infection, proinflammatory cytokines such as TNF‐*α*, IL‐1*β*, and IL‐6 play essential roles in orchestrating the recruitment and activation of effector immune cells. However, excessive or dysregulated production of these mediators can precipitate a cytokine storm, a hyperinflammatory state that exacerbates local tissue injury and drives systemic pathology. This uncontrolled response may escalate into systemic inflammatory response syndrome (SIRS) and can progress to sepsis or multiple organ dysfunction syndrome (MODS), representing severe and often life‐threatening complications of bacterial infection [31, 33].

A cytokine storm can transform what initially serves as an essential antimicrobial defense into a pathological process that paradoxically facilitates bacterial persistence. This hyperinflammatory state not only disrupts host tissue integrity but also diminishes the effectiveness of protective immune responses, ultimately impairing the host′s ability to clear invading pathogens [14, 31]. Under these conditions, subsets of bacteria may adapt to immune and therapeutic pressure through various survival mechanisms, including the formation of biofilms that provide structural protection against antibiotics and phagocytosis, as well as the transition of a portion of the population into metabolically dormant persister cells that exhibit marked tolerance to antimicrobial therapy [34, 35].

Failure to initiate a proper resolution phase in acute inflammation leads to a biologically ineffective, prolonged inflammatory response. This state reshapes the tissue microenvironment in ways that favor bacterial survival, characterized by impaired innate immune function, disorganization of tissue architecture, and a disrupted balance between proinflammatory and proresolutive signals [36]. Collectively, these alterations create a permissive niche that enables bacterial persistence and promotes pathogen adaptation toward chronic infection phenotypes [16, 37].

## 3. Persister Cells as a Biological Link Between Acute Infection and Chronic Disease

Acute bacterial infections that fail to achieve adequate eradication can give rise to subpopulations of cells that adapt by transitioning into a metabolically dormant state [19, 37]. These subpopulations, known as persister cells, exhibit distinct phenotypic features compared with actively replicating bacteria, most notably a profound suppression of metabolic activity. In this dormant state, antibiotic efficacy becomes markedly limited because the primary pharmacological targets of many antimicrobials—including protein synthesis, DNA replication, and cell wall formation—are minimally active or functionally inactive [15].

The persister phenotype arises as an adaptive response to environmental and therapeutic stress, activating a coordinated cellular stress program [19]. This program involves the upregulation of toxin–antitoxin (TA) systems that inhibit translation through ribosomal inactivation or mRNA degradation, alongside the induction of the stringent response driven by the accumulation of (p)ppGpp. These pathways downregulate growth‐associated gene expression and shift bacterial metabolism toward an energy‐conserving state [38].

The decline in ATP availability accompanying this stress response further restricts the enzymatic activities required for cell wall biosynthesis, DNA replication, and protein translation [19, 38]. As a result, antibiotic classes that depend on active bacterial metabolism become markedly less effective. This metabolic shutdown drives bacteria into a reversible dormant state characterized by pronounced pharmacological tolerance [15, 38, 39].

Persister cells typically reside within microenvironments that inherently limit antibiotic penetration, such as hypoxic niches, areas of tissue barrier disruption, sites of unresolved inflammation, and biofilm structures [19, 37]. The combination of these molecular and environmental constraints simultaneously impedes antibiotic distribution to the target bacteria and supports the survival of persistent subpopulations. Consequently, eradication becomes suboptimal, predisposing infections to relapse and chronicity [16].

The presence of persister cells carries significant clinical implications. Although they constitute only a small fraction of the overall bacterial population, these cells are uniquely capable of surviving exposure to conventional antibiotics and serve as a latent reservoir of infection [19]. Once pharmacological pressure is reduced or discontinued, persister cells can undergo reactivation through various resuscitation mechanisms, including nutrient sensing, activation of chemotaxis pathways, and restoration of ribosomal function and protein translation [15]. Through these processes, bacteria regain proliferative capacity, leading to recurrent infection episodes or progression toward more complex and difficult‐to‐manage chronic disease states. Thus, persister cells function as a biological fulcrum connecting unresolved acute inflammation with the establishment of persistent chronic infection, further complicating clinical efforts to overcome antimicrobial resistance [40].

## 4. Biofilm as an Ecological Driver of Chronicity, Relapse, and Immune Failure

Biofilms represent one of the fundamental mechanisms driving the transition of bacterial infections from acute episodes to chronic or recurrent disease states. In this phase, bacteria no longer exist as independent planktonic cells but instead organize into structured communities that adhere to host tissues or medical devices, exhibiting markedly increased tolerance to environmental pressures, including antibiotic exposure and host immune responses [18]. These properties position biofilms as major determinants of infection chronicity and provide a critical explanation for the refractory nature of many persistent bacterial diseases encountered in clinical practice [7].

The resilience of biofilms is largely determined by the presence of an EPS matrix, a highly organized structure composed of polysaccharides, proteins, lipids, and extracellular DNA. This matrix functions not only as a physical barrier that restricts antibiotic diffusion into the microbial community but also creates an internal microenvironment characterized by steep gradients in oxygen availability, nutrient distribution, pH, and accumulated metabolic byproducts [7, 17]. These heterogeneous conditions drive portions of the bacterial population into extremely low metabolic states or near‐nonproliferative phases, rendering many conventional antibiotic targets minimally responsive. The interplay between the EPS matrix and these microenvironmental gradients collectively forms multilayered protective zones, ultimately resulting in uneven bacterial eradication between the superficial layers and the deeper core of the biofilm [39].

Biofilms exhibit a high degree of phenotypic heterogeneity within a single structured community. Rather than forming a homogeneous population, bacteria within biofilms exist as a spectrum of physiological states, including actively proliferating cells, slow‐growing cells, metabolically dormant persister subpopulations that display nongenetic antibiotic tolerance, and viable but nonculturable (VBNC) cells that remain alive yet elude detection by standard culture methods [37, 41]. This heterogeneity carries significant clinical implications, as antimicrobial treatment failure typically does not arise from a single mechanism but from the coexistence of multiple subpopulations with distinct sensitivities to antibiotics and host immune pressures. Moreover, genetically encoded antimicrobial resistance may coexist alongside physiologic tolerance mechanisms characteristic of biofilms and persister cells. Consequently, even in the absence of dominant genetic resistance, biofilm communities can persist through adaptive strategies rooted in metabolic state, growth rate, and microenvironmental conditions [19, 42, 43].

Biofilms constitute highly dynamic systems governed by intricate regulatory networks, including quorum sensing (QS) mechanisms [44, 45]. Through QS, bacteria coordinate the expression of genes involved in virulence, EPS synthesis, and biofilm maturation. QS also plays a pivotal role in orchestrating the dispersal phase, during which a portion of biofilm‐associated cells detach and revert to a planktonic state [46]. These dispersed cells often exhibit enhanced invasive potential, enabling colonization of new sites and triggering acute flare‐ups superimposed on an already established chronic infection. Thus, QS‐mediated regulation and dispersal represent dynamic processes that help explain clinical fluctuations, where periods of relative stability may be followed by acute exacerbations despite apparent control of the primary infection focus [45, 47].

Interactions between biofilms and the host immune system frequently produce a paradoxical state in which persistent inflammation coexists with ineffective pathogen clearance [7, 34]. The biofilm matrix impairs opsonization and phagocytic efficiency while simultaneously inducing repeated but nonresolving neutrophil responses [34, 35]. This results in chronic low‐grade inflammation that contributes to progressive tissue injury without achieving meaningful microbial eradication [34, 35, 42]. In clinical settings such as chronic wounds, biofilms are commonly associated with sustained neutrophil infiltration, the release of reactive oxygen species and proteases, and recurrent cycles of tissue destruction, ultimately expanding and stabilizing the biofilm niche [45, 48].

Collectively, these processes drive a shift in the trajectory of disease, whereby an infection that initially presents as acute can evolve into a chronic or recurrent condition as seen in Figure 1. This transition is characterized by the failure of standard antibiotic regimens to achieve complete eradication, the continued persistence of pathogens within tissue compartments or on the surfaces of implanted medical devices, and an increased likelihood of selecting for bacterial phenotypes with heightened tolerance or even bona fide antimicrobial resistance [16].

**Figure 1:**
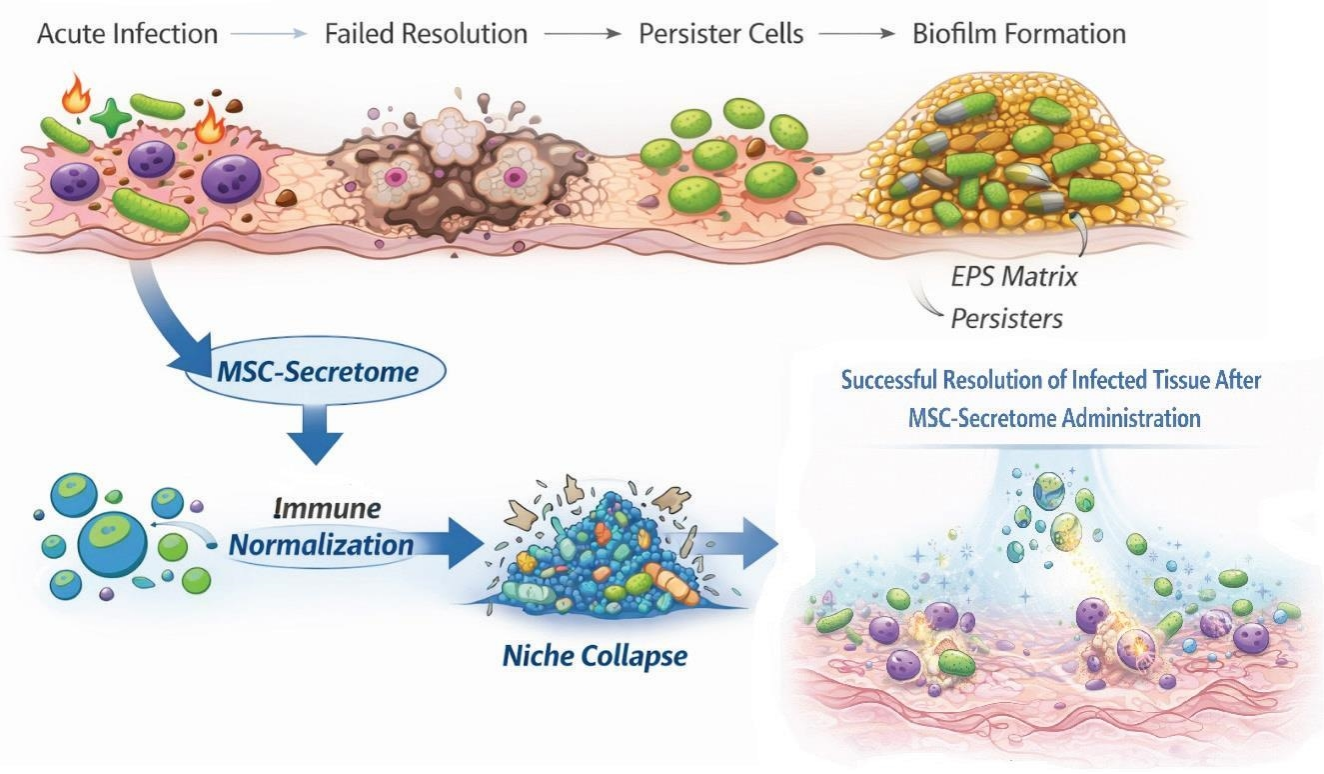
Ecological progression from failed inflammatory resolution to bacterial persistence and biofilm formation. This figure is a conceptual illustration depicting a proposed host–pathogen framework based on integrated evidence from published preclinical and translational studies, illustrating the progression from unresolved acute bacterial infection to the emergence of persister cells and biofilm formation. Color coding and graphical elements: Purple spherical cells represent host innate immune cells (neutrophils and macrophages); green rod‐shaped structures represent Gram‐negative *Bacilli*; yellow‐orange aggregates represent biofilm matrix embedded with EPS; brown necrotic area represents unresolved tissue damage and inflammatory debris; blue spherical cells in the lower panel represent activated macrophages undergoing proresolving reprogramming; green/teal spherical particles represent MSC‐secretome components including extracellular vesicles and soluble mediators. Failure to achieve inflammatory resolution leads to innate immune dysfunction, tissue damage, and the development of an ecological niche that supports bacterial dormancy and stabilizes the EPS matrix. Within this microenvironment, persister cells persist in a metabolically quiescent state, whereas biofilms provide structural protection that further limits antibiotic penetration and immune clearance. MSC‐secretome acts primarily through indirect host‐directed mechanisms to counter these processes. By modulating inflammatory pathways, restoring macrophage function, enhancing tissue repair, and destabilizing biofilm architecture, MSC‐secretome contributes to the re‐establishment of a microenvironment that is less permissive to bacterial persistence and more conducive to pathogen clearance.

## 5. Host–Pathogen Interactions in Persistence: What We Still Do not Understand

Although the concepts of persister cells and biofilms have become central to the discourse on chronic bacterial infections, the detailed host–pathogen interactions that enable these persistent states to endure remain far from fully understood. Emerging molecular studies emphasize that persistence is not a singular phenotype but rather a spectrum of dormancy states of varying depth, continuously shaped by immunological pressures and the surrounding tissue microenvironment [19, 36].

At the bacterial level, one of the most debated domains concerns the role of TA systems. Modules such as HipBA, TisB/IstR, and others have long been viewed as key triggers of persistence due to their ability to suppress protein synthesis, disrupt membrane homeostasis, or reduce ribosomal activity, thereby driving cells into markedly low metabolic states [49]. However, multi‐TA mutant analyses and single‐cell approaches reveal that the removal of numerous TA modules does not consistently decrease persister frequencies, challenging the notion of TA systems as a singular, indispensable mechanism. Instead, current evidence suggests that TA modules represent only one of several potential “entry routes” into dormancy, rather than a universal determinant. Consequently, a definitive genetic framework explaining why only a small subset of bacteria adopt the persister state—despite being exposed to identical stressors—remains elusive [50].

A growing body of work has also introduced new perspectives on the metabolic underpinnings of persistence. Several studies have reported associations between decreased ATP levels, slowed glycolytic and TCA fluxes, and the emergence of energy hysteresis–like phenomenon, in which bacteria remain trapped in a low‐energy metabolic state even after the removal of external stressors, all of which correlate with an increased proportion of persister cells [38, 50]. However, these associations do not clarify causality: Whether ATP depletion actively triggers dormancy, or whether low ATP is simply a downstream consequence of broader metabolic reprogramming initiated by global stress responses, remains unresolved. The complexity deepens in vivo, where bacteria are exposed to gradients of oxygen, nutrients, and immune‐derived reactive oxygen species, making it difficult to distinguish general stress‐adaptation strategies from persister‐specific dormancy programs [16, 51].

This ambiguity becomes even more apparent when positioning persister cells within the context of biofilms. Extensive evidence supports the idea that biofilms function as an “incubator” for persisters: The EPS matrix generates microenvironmental niches characterized by restricted antibiotic diffusion, nutrient limitation, and fluctuating pH, all of which selectively favor subpopulations with markedly reduced metabolic activity [17, 50].

On the other hand, high‐resolution imaging approaches and spatial transcriptomic analyses reveal that even within mature biofilms, bacterial physiological states are highly heterogeneous and dynamically regulated [52, 53]. Subpopulations of cells can transition between active and dormant states in response to fluctuating stress conditions and QS signals. These observations raise an important question: Is the biofilm itself a direct driver of persistence, or is it more accurately viewed as an “ecosystem” that increases the likelihood of persister emergence and maintenance without relying on a single dominant genetic program [41, 54]?

Chronic biofilms in wounds, the respiratory tract, or on implanted medical devices persist not only because of EPS‐mediated protection and antibiotic tolerance but also due to maladaptive crosstalk with the host immune system. Sustained recruitment of neutrophils and macrophages generates inflammatory zones enriched with reactive oxygen species, proteases, and neutrophil extracellular traps (NETs), which paradoxically exacerbate tissue injury while providing matrix fragments and free iron that further support bacterial growth [7, 48]. However, at a mechanistic level, it remains unclear how patterns of immune‐cell recruitment, local cytokine profiles, and failed inflammatory resolution collectively influence the selection of persister subpopulations within biofilms. A key unresolved question is whether persister cells are genuinely “invisible” to the immune system or whether they exploit dysfunctional immune conditions to secure a relatively protected niche [41].

From a clinical standpoint, the long‐standing assumption that persister cells represent a fully reversible dormant state is also being reconsidered. Emerging models propose a graded “dormancy depth,” wherein some persisters occupy shallow dormancy states and readily resuscitate, whereas others exist in profoundly deep dormancy that may require specific host‐derived signals to reactivate [21].

This distinction is clinically important because therapeutic strategies that effectively target shallow persisters—such as “wake‐up” approaches followed by conventional antibiotics—may not be sufficient for deeply dormant subpopulations. At present, no validated in vivo biomarkers exist to differentiate this spectrum of dormancy depth, either at the level of bacterial physiology or within the surrounding host tissue microenvironment [15, 39].

## 6. MSC‐Secretome: Origin, Collection, Composition, and Potential Applications

Mesenchymal stem cells, the biological source of the secretome, can be derived from multiple tissue origins, and this diversity carries meaningful biological consequences. Bone marrow remains the most historically studied source, whereas adipose tissue and umbilical cord have gained increasing attention due to easier procurement and reduced susceptibility to donor age‐related changes in cell quality [55]. Other sources—including dental pulp, Wharton′s jelly, and placenta—have also been described, though their secretome profiles remain less well‐characterized [55, 56]. Importantly, tissue origin is not merely a logistical variable; it meaningfully shapes the immunomodulatory and regenerative output of the resulting secretome, and the choice of source should ideally reflect the intended therapeutic application [56].

Secretome collection is typically performed through a conditioned medium protocol. MSCs are cultured to 60%–80% confluency, washed with phosphate‐buffered saline to minimize serum carryover, and then incubated in serum‐free medium for 24–48 h before harvest. The collected medium undergoes sequential centrifugation to remove debris, followed by ultrafiltration or size‐exclusion chromatography for concentration [56]. Complete elimination of residual serum proteins remains technically difficult, and this contamination—even at trace levels—complicates characterization and limits cross‐study comparability. Storage at −80°C is standard, though lyophilization is increasingly explored as a more scalable alternative for clinical translation [56, 57].

The MSC‐secretome comprises two principal fractions: a soluble component containing cytokines and growth factors such as IL‐10, TGF‐*β*, PGE2, HGF, and VEGF, and a vesicular component—predominantly exosomes—carrying regulatory microRNAs including miR‐146a, miR‐21, and miR‐223 [58, 59]. Characterization approaches range from mass spectrometry and cytokine arrays to RNA sequencing. However, the compositional profile is not static; it varies substantially depending on tissue source, donor characteristics, culture conditions, and preconditioning strategies. Batch‐to‐batch variability and the absence of standardized quality control parameters remain among the most pressing unresolved challenges in the field [58, 59].

The therapeutic applications of MSC‐secretome span a wide range of pathological contexts [24]. In infectious and inflammatory diseases—the primary focus of this review—it offers a host‐directed strategy proposed to restore immune homeostasis and potentially disrupt the microenvironmental conditions that sustain bacterial persistence and biofilm formation. Beyond infection, preclinical and early clinical data support its potential in acute lung injury, chronic wound healing, liver fibrosis, and neuroinflammatory conditions [60, 61]. Compared with cell‐based therapies, the secretome offers practical advantages in terms of storage, handling, and safety profile, making it a promising candidate for clinical development—provided that the outstanding challenges of standardization, delivery optimization, and regulatory classification are adequately addressed [60, 62, 63].

## 7. From Antibiotic Eradication to Secretome‐Based Host‐Directed Control of Bacterial Persistence and Biofilm‐Associated Infections

Therapeutic approaches that focus solely on pathogen eradication through antibiotics are often insufficient to disrupt the dormancy–reactivation cycle that underlies recurrent and chronic bacterial infections. Although appropriate antibiotic selection and optimized tissue exposure based on pharmacokinetic and pharmacodynamic principles remain essential components of initial management, sublethal drug concentrations can still promote the early emergence of tolerant bacterial phenotypes [21, 36]. For this reason, early management must be conceptualized as a comprehensive eradication strategy—one that not only targets the pathogen directly but also closes the biological “escape routes” that enable persistence.

Beyond antimicrobial therapy, early and adequate source control—including abscess drainage, debridement of necrotic tissue, and removal of colonized medical devices—represents a critical pillar in preventing infection chronicity. Tissue microenvironments enriched with biofilm, hypoxia, acidic pH, and high bacterial burden create protective niches that sustain persister subpopulations and diminish antibiotic efficacy [15]. Within this context, emerging insights into persister biology underscore that long‐term therapeutic success requires interventions directed at the foundational mechanisms of persistence, including metabolic dysfunction, the stringent response, TA systems, and the structural protection afforded by biofilms [38, 40].

Within this framework, the MSC‐secretome can be positioned as a host‐directed therapeutic approach that addresses the biological conditions underlying the transition from unresolved acute inflammation to chronic, biofilm‐associated disease states driven by persister cell persistence [20]. Unlike conventional strategies that act directly on the pathogen, host‐directed approaches aim to modulate the immune response and restore tissue microenvironmental homeostasis, thereby eliminating the conditions that sustain persistent bacterial populations [22, 64]. Effective inflammatory resolution accompanied by restored phagocyte function increases host‐based biological pressure, enabling more efficient bacterial clearance without contributing to antibiotic‐driven selective pressure [62, 65].

Mechanistically, MSC‐secretome may modify host and tissue microenvironmental conditions that support the survival of persistent bacterial subpopulations through pathways centered on inflammatory resolution and restoration of innate immune function—effects that have not yet been directly quantified in terms of persister frequency or dormancy depth. This effect is mediated by the downregulation of key proinflammatory cytokines—such as TNF‐*α*, IL‐1*β*, and IL‐6—alongside the enhancement of anti‐inflammatory and proresolutive mediators including IL‐10, TGF‐*β*, and prostaglandin E2. Together, these changes improve phagocyte function and promote tissue repair [24, 26, 66]. Reprogramming of macrophages and monocytes toward a proresolving phenotype—shifting from M1 to M2 polarization—helps maintain phagocytic capacity and immune‐metabolic function, while also enhancing endogenous antimicrobial peptide production and intracellular antibacterial effectors. These concerted actions may contribute to more sustained elimination of persistent bacterial populations [25, 64].

In methicillin‐resistant *Staphylococcus aureus* (MRSA) infections, MSC‐derived secretome has demonstrated direct antibiofilm activity. Its cysteine protease components, particularly cathepsin B and cathepsin V, can destabilize the extracellular matrix of established biofilms, reducing their mass and structural integrity while enhancing antibiotic penetration and effectiveness [20, 67]. In addition, MSC‐secretome contains antimicrobial and matrix‐regulatory factors such as cystatin C, elafin, lipocalin, and cathelicidin (LL37), all of which contribute to suppression of bacterial growth [68, 69]. Lipoxin A4 (LXA4) activates the ALX/FPR2 receptor on macrophages, promoting a proresolving functional state that enhances phagocytic clearance of bacteria sequestered within biofilm‐associated niches [70]. By disrupting the protective biofilm niche, MSC‐secretome may reduce microenvironmental conditions that favor dormant and antibiotic‐tolerant bacterial states, potentially increasing susceptibility to immune‐mediated clearance as well as conventional antimicrobial therapies, without amplifying antibiotic selective pressure [23, 28, 71].

At the molecular level, MSC‐derived secretome modulates the inflammatory balance toward resolution by suppressing NF‐*κ*B activation, a process largely mediated by exosomal microRNAs (such as miR‐146a) that directly target IRAK1 and TRAF6, while concurrently engaging immunoregulatory pathways involving STAT3 and PPAR‐*γ*. Activation of these pathways enhances the production of proresolving mediators, optimizes efferocytosis, and restores tissue homeostasis [30, 58, 72]. A range of bioactive components within MSC‐secretome—including PDGF‐AA, endothelin‐1, IGFBP‐2, and extracellular matrix elements such as tenascin‐C and PAI‐1—act synergistically to promote microangiogenesis, improve tissue oxygenation, and accelerate the resolution and maturation of granulation tissue during wound repair. By normalizing the microenvironment, these effects may collectively dismantle the ecological conditions that support biofilm stability and bacterial persistence [68, 71, 73].

## 8. Preclinical Evidence Supporting MSC‐Secretome in Bacterial Infections

Several preclinical studies provide direct support for the antibacterial and antibiofilm effects of MSC‐secretome discussed above. In an equine MRSA model, bone marrow MSC‐secretome reduced established biofilm mass through cysteine protease activity, with cathepsin B and cathepsin V identified as the main effectors [20]. In a murine model of MRSA‐infected skin wounds, topical secretome application accelerated wound closure and increased macrophage infiltration compared with untreated controls [68], a finding consistent with in vitro data showing measurable antimicrobial activity of adipose‐derived MSC secretome against MRSA [74].

Beyond localized infection, secretome administration in a rat sepsis model reduced circulating proinflammatory cytokines and improved survival, indicating that its immunomodulatory effects extend beyond the local site of injury [66]. Secretome from dental pulp MSCs has likewise shown inhibitory activity against several clinically relevant bacterial strains [23].

Together, these animal and in vitro findings—spanning equine, murine, and human models—point to two complementary modes of action: direct antibiofilm/antimicrobial activity and indirect host‐directed immunomodulation. Clinical trial data specifically evaluating MSC‐secretome for bacterial infections in humans are currently absent, and this represents a critical knowledge gap that future translational research must address. The complementary host‐directed and antimicrobial/antibiofilm mechanisms discussed above are summarized in Table 1.

**Table 1:** Mechanistic summary of MSC‐derived secretome in bacterial persistence and biofilm‐associated infections.

Mechanism level	Key biological targets	MSC secretome components/processes	Key mechanistic effects	Implications for persistence and biofilms
Tissue microenvironment (ecological niche) [26, 28, 68]	Hypoxia, acidic pH, damaged tissue, chronic inflammation	Vascular proregenerative factors, inflammatory resolution mediators, PDGF‐AA, endothelin‐1, IGFBP‐2	Normalization of micro‐oxygenation, improvement of vascularization, restoration of tissue integrity	Reduction of ecological conditions that favor bacterial dormancy and biofilm stability
Innate immunity [25, 58, 68]	Macrophages, monocytes, neutrophils	Immune‐metabolic reprogramming; through increased IL‐10, PGE2, TGF‐*β*	Phenotype shift of macrophages M1 → M2 (resolutive)	Proposed restoration of phagocytic capacity, potentially improving host‐mediated clearance of persistent bacterial subpopulations
Phagocyte function [70, 75]	Phagocytosis, intracellular killing	Activation of intracellular antimicrobial effectors, endogenous antimicrobial peptides; LL‐37, lipoxin A4	Proposed enhancement of phagocytic clearance of nonreplicative and antibiotic‐tolerant bacterial subpopulations	Potential reduction in the reservoir of persistent bacterial subpopulations that sustain chronic infection cycles
Biofilm matrix [20, 71]	Biofilm EPS (protein and structural matrix)	Protease (cathepsin B, cathepsin V)	Destabilization of the biofilm extracellular matrix	Decreased biofilm mass and stability; increased antibiotic efficacy against biofilm‐embedded bacteria
Bacterial metabolic dormancy [23, 67, 70]	Persister cells in biofilm niche	Niche destabilization, host immune pressure; LL‐37, lipoxin A4	Proposed interference with dormant and tolerant bacterial states	Potentially increased susceptibility to immune‐mediated clearance and antibiotic treatment
Resolution and immunoregulation signaling [62, 64, 66]	STAT3, PPAR‐*γ*	Activation of immunoregulatory pathways through increased IL‐10, PGE2, TGF‐*β*	Increased efferocytosis and tissue homeostasis	Sustained resolution of inflammation
Molecular signaling of inflammation [30, 32, 73]	NF‐*κ*B and proinflammatory cytokines TNF‐*α*, IL‐1*β*, IL‐6	Suppression of NF‐*κ*B activation pathway via IL‐10, PGE2, TGF‐*β*, extracellular vesicle–associated miR‐146a	Decreased expression of proinflammatory genes and Transition from persistent inflammation to resolution phase	Potentially reducing chronic inflammation that maintains cell persistence and tissue tolerogenicity
Antibiotic selective pressure [22, 23, 67, 69]	Sublethal exposure to antibiotics	Indirect effects (host‐directed) and the ability to inhibit bacteria by the content of cystatin c, elafin, lipocalin, cathelicidin	May exert less direct antimicrobial selective pressure than conventional antibiotics	Its effects on the long‐term evolution of bacterial tolerance, resistance, and recurrence remain insufficiently investigated

Overall, the principal advantage of MSC‐derived secretome does not lie in any direct bactericidal activity, but rather in its ability to function as an ecological modulator that restores the integrity of the local tissue microenvironment and rebalances dysregulated host immune responses. By reducing tissue tolerogenicity, enhancing phagocyte competency, and weakening the architectural stability of established biofilms, the secretome positions itself as a host‐directed therapeutic modality capable of interrupting the transition from unresolved acute inflammation to chronic, biofilm‐associated infections maintained by persister cells as seen in Figure 2 [24].

**Figure 2:**
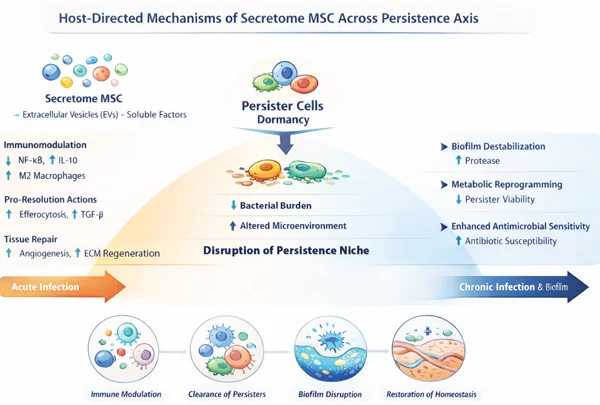
Host‐directed modulation of immune and tissue microenvironments by MSC‐derived secretome across the bacterial persistence axis.

This conceptual schematic summarizes experimentally demonstrated and mechanistically proposed actions of MSC‐derived secretome at the host–pathogen interface and does not represent findings from a single experimental system. At the host level, MSC‐secretome modulates excessive NF‐*κ*B signaling, promotes proresolving macrophage responses, enhances efferocytosis, and supports restoration of immunologic balance. Within the tissue microenvironment, secretome‐associated regenerative and proresolving factors contribute to angiogenesis, extracellular matrix repair, and restoration of tissue homeostasis. On the microbial side, preclinical evidence supports antimicrobial and antibiofilm activities, including biofilm matrix destabilization, whereas direct effects on persister frequency, dormancy, or persister clearance remain insufficiently demonstrated. Collectively, these host‐directed and antibiofilm effects may reduce microenvironmental conditions that favor bacterial persistence and potentially enhance susceptibility to immune‐mediated clearance and conventional antimicrobial therapy.

## 9. Limitations, Standardization, and Translational Perspectives

To date, there is no widely accepted framework for the standardization of MSC‐secretome production, characterization, or quality control, including the definition of biologically relevant potency parameters for applications targeting bacterial persisters and biofilm‐associated infections [56]. The biological heterogeneity of the secretome remains a major challenge, as it is strongly influenced by the tissue source of MSCs—such as bone marrow, adipose tissue, or umbilical cord—as well as donor‐related characteristics, isolation methodology, and culture conditions. Variations in environmental cues, particularly normoxic versus hypoxic priming, further shape the composition of bioactive mediators, including cytokines, growth factors, lipid‐derived proresolving molecules, and microRNAs. These differences collectively lead to substantial batch‐to‐batch variability in the biological effects of the resulting secretome [56].

The optimal dosing strategy for MSC‐derived secretome in infectious diseases has not yet been defined. Therapeutic outcomes appear to reflect the complex interplay between secreted bioactive mediators, the host immune system, and the surrounding tissue microenvironment, rather than a simple linear relationship between administered dose and systemic concentrations [76]. In addition to unresolved dosing parameters, several other challenges remain, including the selection of an appropriate delivery route, the evaluation of short‐ and long‐term safety, and the establishment of a clear regulatory pathway for clinical translation [77].

Intravenous administration is limited by the rapid first‐pass clearance of extracellular vesicles and soluble mediators by the reticuloendothelial system, which may shorten effective exposure time at target tissues [78, 79]. Nonintravenous delivery methods, such as intramuscular injection, can provide a more gradual and sustained release of bioactive factors. However, comparative studies assessing the relative effectiveness of different administration routes specifically in the context of bacterial infections remain scarce [63, 80].

Another major limitation is the predominance of acute or subacute models; MSC‐derived secretome is rarely evaluated in experimental systems that truly recapitulate long‐term chronicity, such as orthopedic implant–associated biofilms or persistent infections seen in advanced bronchiectasis [54, 61]. Furthermore, substantial variability in MSC tissue origin, preconditioning strategies, and the relative contributions of soluble versus extracellular vesicle fractions complicates efforts to compare antipersister effects across studies. Although some reports describe robust antimicrobial or antibiofilm activity, these findings often rely on laboratory‐adapted strains or a narrow range of clinical isolates. As a result, it remains uncertain whether such effects are broadly applicable or highly strain‐specific [74].

Methodological limitations are further compounded by the scarcity of physiologically relevant experimental platforms for evaluating secretome activity in chronic infection contexts. Standard two‐dimensional culture systems and planktonic bacterial models fail to recapitulate the structural complexity, oxygen gradients, and EPS heterogeneity characteristic of established biofilms. Although three‐dimensional biofilm models and organ‐on‐chip platforms that incorporate host immune cells and tissue microenvironments have been developed and applied to antimicrobial research, their integration into secretome‐based studies remains limited [81]. Similarly, although miRNA fingerprinting approaches—including miR‐146a, miR‐21, and miR‐223 profiling—and functional potency assays targeting EPS degradation or efferocytosis enhancement have been proposed as standardization tools for MSC‐secretome characterization, validated release criteria based on these markers do not yet exist for secretome products intended for infectious disease applications [56, 59].

The safety profile of MSC‐derived secretome is generally more favorable than that of living cell–based therapies; however, several potential risks still warrant careful consideration. These include the possibility of excessive immunomodulation, interference with protective phases of acute inflammation, and unintended proangiogenic or profibrotic effects [82]. From a regulatory standpoint, secretome occupies an intermediate position between biological products, cell‐derived therapeutics, and advanced therapy medicinal products (ATMPs), categories for which harmonized regulatory frameworks remain incomplete [83]. Collectively, these challenges underscore that the clinical translation of secretome will require biological and clinical evaluation strategies distinct from those applied to conventional pharmacological agents.

## 10. Future Directions: What Should the Field Do Next?

Although preclinical findings increasingly suggest that MSC‐derived secretome holds promise as a host‐directed therapeutic approach for addressing bacterial persistence and biofilm‐associated infections, the field still requires a more systematic research framework before meaningful clinical translation can be realized [22]. A major limitation lies in the experimental platforms currently in use. Most studies continue to rely on 2D culture systems that fail to capture the metabolic heterogeneity, oxygen gradients, and EPS complexity characteristic of chronic biofilms. Recent advances have produced several promising experimental platforms, including implantable multispecies biofilm models, perfused organ‐on‐chip systems incorporating host immune cell compartments, and ex vivo wound infection models that partially replicate hypoxic gradients and sustained inflammatory burden [81, 84]. These tools have demonstrated utility in antimicrobial research more broadly, enabling spatial and temporal resolution of bacterial physiological states within biofilm communities. However, none of these platforms has yet been systematically applied to evaluate MSC‐secretome activity under conditions that faithfully recapitulate long‐term chronicity or persister–biofilm dynamics, making it difficult to draw firm mechanistic conclusions from existing studies. Consequently, adaptation, validation, and systematic application of these advanced platforms to MSC‐secretome research are needed to evaluate secretome activity under more physiologically relevant and chronic infection conditions [81, 84].

Beyond improved models, the field also requires biomarkers capable of objectively assessing the multifaceted effects of secretome. A composite biomarker panel integrating indicators of inflammation resolution (e.g., IL‐10, TGF‐*β*, and M1‐to‐M2 macrophage transitions) [25, 66], biofilm destabilization markers (extracellular DNA, polysaccharide intercellular adhesin [PIA] [47], and QS autoinducers), and measures of tissue integrity would be invaluable for determining whether interventions meaningfully alter the tolerogenic microenvironment that sustains bacterial persistence. Without such biomarkers, evaluations of secretome will remain largely descriptive and difficult to compare across studies [44, 45, 60].

At the same time, the need for biological standardization cannot be postponed [56]. Batch‐to‐batch variability in secretome preparations remains substantial and poses a major obstacle to reproducibility. MicroRNA fingerprints—such as miR‐146a, miR‐21, or miR‐223—may serve as potential product identity markers, whereas functional potency assays that evaluate EPS degradation, enhancement of efferocytosis, or increased susceptibility of persister cells should be further validated as biologically meaningful release criteria [59].

Translational considerations also require clearer guidance regarding the preferred route of administration. For chronic infections characterized by a dysfunctional tissue microenvironment, intramuscular or locally directed delivery appears more promising than intravenous infusion, which is prone to rapid reticuloendothelial clearance [77, 78]. Finally, combining secretome with antibiotics—particularly through sequential regimens designed to disrupt biofilm architecture before antimicrobial exposure—represents a highly rational synergistic strategy that warrants systematic investigation [71, 80].

Overall, future research efforts should advance toward more physiologically relevant models, quantifiable and mechanism‐informed biomarkers, rigorous standardization frameworks, optimized delivery routes, and rational combination strategies. Within such an integrated framework, MSC‐derived secretome can be evaluated more appropriately as an adjuvant therapeutic option for persistent bacterial infections and biofilm‐associated disease.

## 11. Conclusion

The failure to eradicate bacterial infections does not merely reflect the limitations of antimicrobial agents, but also the inability of the host immune system to achieve effective inflammatory resolution. When the resolution phase is incomplete, the tissue microenvironment shifts toward a tolerogenic state that creates an ecological niche conducive to the emergence of persister cells and the stabilization of biofilm structures. Under these conditions, therapeutic strategies that rely solely on bactericidal activity are often insufficient to disrupt the cycles of persistence and recurrent infection. Within this context, MSC‐derived secretome represents a promising host‐directed approach, acting through indirect mechanisms that may restore innate immune function, promote inflammatory resolution, and normalize disrupted tissue microenvironments. Consistent preclinical evidence across diverse infection models supports the concept that modulating host ecology may reduce bacterial tolerance, enhance antibiotic susceptibility, and improve structural and immunologic integrity within affected tissues.

The strategic advantage of MSC‐derived secretome lies in its potential to support the host′s intrinsic biological ability to control infection in a more stable and sustained manner, thereby reducing reliance on increasingly intensive antibiotic regimens. Nonetheless, several knowledge gaps continue to impede clinical translation. Significant batch‐to‐batch biological variability and the absence of objective biomarkers capable of capturing secretome‐induced biofilm modulation or inflammatory resolution indicate that a robust standardization framework is still lacking. Methodologically, the scarcity of preclinical models that faithfully replicate persister–biofilm dynamics and their interactions with host tissues limits the generalizability of existing findings. From a clinical perspective, the potential of MSC‐derived secretome as an adjuvant therapy for chronic infection must be balanced with rigorous long‐term safety evaluation, careful determination of the optimal delivery route, and a clearer regulatory pathway for complex biological products.

Accordingly, more systematic and mechanistically informed research is essential. This includes the application and validation of physiologically relevant 3D biofilm and organ‐on‐chip models for MSC‐secretome studies, the establishment of miRNA fingerprints and functional potency assays as part of the release criteria, and the exploration of mechanism‐based secretome–antibiotic combination regimens. These efforts will not only strengthen the scientific foundation of MSC‐derived secretome therapy, but also serve as critical prerequisites for its safe and effective translation into clinical practice for the management of persistent bacterial infections and biofilm‐associated diseases.

## Funding

No funding was received for this manuscript.

## Conflicts of Interest

The authors declare no conflicts of interest.

## Data Availability

Data sharing is not applicable to this article as no datasets were generated or analyzed during the current study.
